# A randomized controlled trial using surgical gloves to prevent chemotherapy-induced peripheral neuropathy by paclitaxel in breast cancer patients (AIUR trial)

**DOI:** 10.1186/s12885-023-11079-8

**Published:** 2023-06-20

**Authors:** Young-Joon Kang, Chang Ik Yoon, Yun-Jung Yang, Jong Min Baek, Yong-Seok Kim, Ye Won Jeon, Jiyoung Rhu, Jae Pak Yi, Dooreh Kim, Se Jeong Oh

**Affiliations:** 1 Department of Surgery, Incheon St. Mary’s Hospital, The Catholic University of Korea, 56, Dongsu-Ro, Bupyeong-Gu, Incheon, 21431 South Korea; 2 Department of Surgery, Seoul St. Mary’s Hospital, The Catholic University of Korea, Seoul, Korea; 3 Department of Convergence Science, College of Medicine, Catholic Kwandong University, Incheon, Korea; 4 Department of Surgery, Yeouido St. Mary’s Hospital, The Catholic University of Korea, Seoul, Korea; 5 Department of Surgery, Uijeongbu St. Mary’s Hospital, The Catholic University of Korea, Uijeongbu, Korea; 6 Department of Surgery, St. Vincent’s Hospital, The Catholic University of Korea, Suwon, Korea; 7 Department of Surgery, Bucheon St. Mary’s Hospital, The Catholic University of Korea, Bucheon, Korea

**Keywords:** Breast neoplasm, antineoplastic agents, Adverse effects, peripheral nervous system disease, Chemically induced

## Abstract

**Background:**

Chemotherapy-induced peripheral neuropathy (CIPN) is a common adverse effect of taxane treatment and can significantly affect patient quality of life. Currently, there are no effective treatments to alleviate symptoms of CIPN; thus, starting with prevention steps in high-risk patients is considered advantageous. However, for these prevention steps to be applicable to all patients, their side effects or accompanying discomforts should be minimal, and the intervention cost-effective. Compression therapy can be considered a prevention intervention, and using surgical gloves is feasible and cost-effective (approximately $0.6 per pair). Although previous studies on compression therapy using surgical gloves have reported decreased incidence of PN, these studies were non-randomized, limited to nab-paclitaxel treatment, and involved the use of small gloves, which may have caused discomfort. Therefore, this study aimed to assess the preventive effects of compression therapy using normal-sized surgical gloves on CIPN in patients treated with paclitaxel.

**Methods:**

This clinical trial is designed to evaluate the preventive effects of compression therapy using surgical gloves on CIPN in women with stage II–III breast cancer who received paclitaxel chemotherapy for at least 12 weeks. This multicenter, randomized-controlled, open-label study will be conducted in six academic hospitals. Patients with medication or a medical history related to neuropathy or hand disease will be excluded. The primary outcome will be the preventive effect of compression therapy using surgical gloves, measured based on changes in the neurotoxicity component of the Functional Assessment of Cancer Therapy-Taxane questionnaire. Furthermore, we will assess the National Cancer Institute’s Common Terminology Criteria for Adverse Events grade of CIPN after 6 months. Notably, the estimated sample size, based on a *p*-value < 0.025 and statistical power of 0.9, will consist of 104 patients (52 per group), accounting for a 10% sample loss.

**Discussion:**

This intervention can be easily implemented in clinical practice and may serve as a preventive strategy for CIPNs with strong patient adherence. If successful, this intervention could improve the quality of life and treatment adherence in patients receiving chemotherapy that can induce PN, extending beyond paclitaxel treatment alone.

**Trial registration:**

ClinicalTrials.gov, NCT05771974; Registered on March 16, 2023.

**Supplementary Information:**

The online version contains supplementary material available at 10.1186/s12885-023-11079-8.

## Background

Chemotherapy-induced peripheral neuropathy (CIPN) is a common adverse effect of taxane-based treatments that affects up to 60% of patients undergoing chemotherapy [1–3]. The occurrence of CIPN is anticipated to increase owing to increased survival rates [4], which significantly affect patient quality of life (QoL) and often lead to early termination of treatment [5]. According to the American Society of Clinical Oncology (ASCO) guidelines, duloxetine is the only treatment agent that can be recommended for CIPN; however, it has moderate strength [6, 7]. At present, there are no effective treatments to alleviate symptoms of CIPN and no agents or interventions to prevent CIPN. However, it is considered advantageous to start with prevention steps that can be widely applied to high-risk patients. Nevertheless, for these prevention steps to be applicable to all patients, their side effects or accompanying discomforts should be minimal and the interventions cost-effective.

To achieve this, we considered preventive methods with no more than “moderate harm” whose “strength of recommendation” was not “against” in ASCO guidelines, and “grades of recommendation” was not “D” or “E” in the European Society for Medical Oncology (ESMO) guidelines. In ASCO guidelines, the “strength of recommendation” for all preventive medications, except ganglioside-monosialic acid (GM)-1, is “moderate or strong against” [7]. On the other hand, ESMO guidelines showed no positive recommendation for pharmacological prevention [6]. In addition, interventions such as acupuncture, cryotherapy, compression therapy, and exercise therapy have been considered for the prevention of CIPN. Among them, acupuncture and cryotherapy have moderate harm, but their definitive recommendations remain scarce. In particular, some studies have indicated that compression therapy using surgical gloves can decrease the incidence of PN [8, 9]; however, these studies were limited by their small sample sizes, non-randomized designs, and a focus on nab-paclitaxel. Likewise, a prior study, reported that using small gloves may cause patient discomfort [10]. Moreover, even though cryotherapy is as effective as compression therapy, it requires complex preparation and has a high discontinuation rate due to discomfort [11].

Nevertheless, because preventive interventions should ideally be readily available, cost-effective, and have high patient adherence, we consider that surgical gloves offer a potential solution that is both practical and cost-effective (approximately $0.6 per pair). Therefore, this study aimed to examine the impact of wearing normal-sized surgical gloves on CIPN prevention to confirm the findings of a previous study that reported no difference in efficacy between small and normal-sized gloves [12]. In addition, the survival rates of patients with breast cancer as improved, as the number of women who died of breast cancer has reportedly decreased by 43% in 2020, following the peak recorded in 1989 in the United States [13] and by approximately two-fifths (41%) in the United Kingdom since the early 1970s [14]; thus, there has been a growing focus on their QoL [15, 16]. However, previous studies on CIPN have often depended on investigator judgment [17–19], causing clinicians to underestimate patient-reported symptoms [20]. Moreover, the significance of patient-reported outcomes has recently increased, with many clinical trials incorporating them as study endpoints [21–24]. Besides, enhanced survival has been linked to patient-reported symptom monitoring in patients with metastatic cancer [25, 26]. Therefore, this study will comprehensively explore the QoL of patients with breast cancer.

Furthermore, we also aim to establish whether compression therapy using two normal-sized surgical gloves in patients receiving paclitaxel treatment could decrease the incidence of CIPN, by comparing the results from the Functional Assessment of Cancer Therapy—Neurotoxicity (FACT-NTX) component questionnaire before and after neoadjuvant/adjuvant chemotherapy with paclitaxel in patients with breast cancer. Lastly, this study will investigate changes in the FACT-Taxane and National Cancer Institute’s Common Terminology Criteria for Adverse Events (NCI-CTCAE) grade for CIPN after 6 months of chemotherapy.

## Methods/Design

### Ethics statement

The study protocol has been approved by the Institutional Review Board (IRB) of Catholic Medical Center (IRB number: XC23DIDS0001). Patient informed consent will be obtained by the co-investigators authorized by the IRB of each participating center. An electronic case report form will be used to collect data that will ensure patient de-identification. The trial has been registered with clinicaltrials.gov (NCT05771974; date of registration, March 16, 2023) and Clinical Research Information Service (KCT0008371; date of registration, April 6, 2023).

### Study design

The primary purpose of this article is to outline the design and methodology of our randomized controlled trial aimed at determining the efficacy of compression therapy using two normal-sized surgical gloves in reducing CIPN incidence in patients receiving paclitaxel treatment. Six academic institutions in South Korea will participate in this study.

Enrolled patients will undergo treatment for breast cancer with either 175 mg/m^2^ of paclitaxel over 3 h via an intravenous (IV) infusion every 3 weeks for four cycles or 80 mg/m^2^ of paclitaxel over 3 h via IV infusion every week for 12 cycles; this is the regimen for neoadjuvant or adjuvant chemotherapy. Human epidermal growth factor receptor 2-positive patients with breast cancer will receive trastuzumab at an initial dose of 8 mg/kg and a loading dose of 6 mg/kg via IV infusion every 21 d. In addition, trastuzumab will be injected weekly at 4 mg/kg, with the first dose being paclitaxel, followed by 2 mg/kg IV weekly to complete the paclitaxel regimen. Alternatively, subcutaneous administration of trastuzumab is possible. Importantly, because of the rules set forth by the national insurance system in South Korea, dose-dense regimens will not be used in this study.

### Study participants

Participants will undergo screening tests to assess their eligibility according to the inclusion and exclusion criteria below.

The inclusion criteria are as follows:

- 1. Patients aged ≥ 19 years but ˂ 70 years
- 2. Those with stages II–III breast cancer
- 3. Those without distant metastasis
- 4. Those scheduled to be receiving adjuvant or neoadjuvant paclitaxel for at least 12 weeks
- 5. Those who provided informed consent
- 6. Those with an Eastern Cooperative Oncology Group performance status ≤ 2 (Karnofsky ≥ 60%)

The exclusion criteria are as follows:

- 1. Patients with recurrent breast cancer
- 2. Those who previously received treatment that could cause neuropathy, e.g., taxane- or platinum-based chemotherapy and anti-tubulin and proteasome inhibitor treatments
- 3. Those with a history of neuropathy
- 4. Those with chronic kidney disease
- 5. Those with the Raynaud phenomenon
- 6. Those with peripheral vascular disease or peripheral arterial ischemia
- 7. Those with cold intolerance
- 8. Those with an allergy to natural rubber, latex, or surgical gloves
- 9. Those who have dermatopathy, wound, or musculoskeletal problems of the hands at enrollment
- 10. Those taking current medications that may mitigate CIPN, including duloxetine; gabapentin/pregabalin; topical amitriptyline, ketamine, baclofen; oral cannabinoids; tricyclic antidepressants; and ganglioside-monosialic acid [7].

### Intervention

In the intervention group, each patient will be required to wear two normal-sized surgical gloves on each hand. Since surgeons use surgical gloves that fit snugly on their hands, a normal-sized surgical glove is defined as a surgeon's fit. The gloves will be worn 30 min before the administration of paclitaxel, throughout paclitaxel infusion, and for 30 min after the infusion. No additional interventions will be applied except for the surgical gloves. Notably, surgical gloves should fit the patients’ hands. The attending surgeons in this study will determine the appropriate glove size for each patient. They will place their palm against each patient’s palm and estimate the glove size that best fits each patient’s hand. Particularly, samples of every glove size will be prepared and confirmed for each patient by the surgeons before the intervention begins. We plan to standardize the use of one glove manufacturer, namely Protexis Latex Essential (Cardinal Health, IL, USA). In contrast, in the control group, patients will not wear surgical gloves or other hand coverings during the paclitaxel infusion.

### Assessment measures

**Fig. 1:**
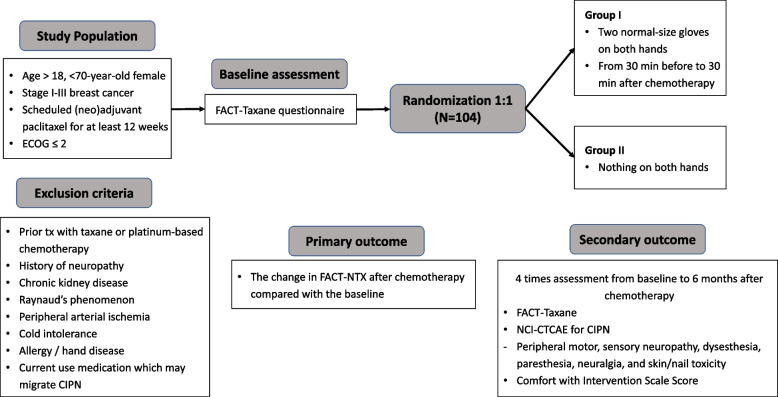
AIUR trial: Study design

The primary outcome will be the percentage of patients with a significant decrease in FACT-NTX scores before the paclitaxel course (baseline) and after the paclitaxel chemotherapy course (target) [27] (Fig. 1). FACT-NTX is an 11-item tool used to evaluate symptoms and concerns specifically associated with chemotherapy-induced neuropathy. FACT-Taxane is a 5-point Likert-type scale, with each item measured on a 0 – 4 scale (0, not at all; 4, very much). A higher FACT-NTX score indicates a worsening of PN in the recent version 4 (Supplement 1). A 5-point or greater change (i.e., > 10% change) in the FACT-NTX score will be considered a clinically meaningful increase in CIPN incidence [28, 29]. In this study, changes in FACT-NTX scores will be divided into a good (change in FACT-NTX < 5 points from baseline to the target timeframe) or a poor (change in FACT-NTX ≥ 5 points from baseline to the target timeframe) outcome, and differences in the number of participants with poor outcomes will be compared between the two groups.

**Fig. 2:**
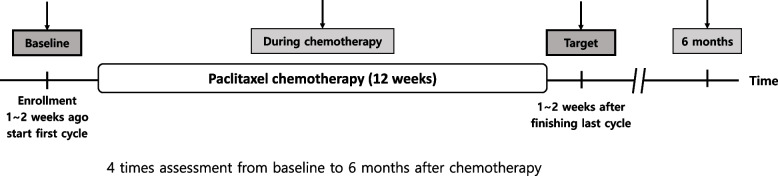
Diagram for the study time process

The time frame will be divided into four periods (Fig. 2). Notably, the baseline is set to be assessed from 1 to 2 weeks before paclitaxel chemotherapy and immediately before the infusion. During paclitaxel treatment, patients will be assessed in the middle of the schedule i.e., 1–2 weeks after the second cycle in the 3 weeks schedule and after the sixth cycle in the weekly schedule. The target timeframe is 1 and 2 weeks after completing all paclitaxel cycles, and patients will be assessed after the last evaluation, which is 6 months from the last paclitaxel cycle.

FACT-Taxane is a self-report instrument developed to measure the health-related QoL of patients receiving taxane-containing chemotherapy. FACT-Taxane comprises FACT-General (FACT-G) [30] and a 16-item taxane subscale. FACT-G comprises subscales assessing physical, social/family, emotional, and functional well-being. The Taxane subscale combines the 11-item neurotoxicity subscale with five additional questions assessing symptoms related to arthralgia, myalgia, and skin discoloration.

The secondary outcomes will be changes in FACT-Taxane and NCI-CTCAE scores for CIPN at baseline, during chemotherapy, at the target time frame, and 6 months after completion of the paclitaxel course. The NCI-CTCAE version 5.0 for CIPN is a method for reporting the severity of adverse events using a combination of clinical evaluations. These adverse events include peripheral motor function, sensory neuropathy, dysesthesia, paresthesia, neuralgia, and skin/nail toxicity. Participants will also be checked for comfort using the intervention scale score during the course of paclitaxel, at the target timeframe, and 6 months later.

The participants will not proceed with self-reporting alone, as the attending physician or research nurse will always assist the patients with the questionnaire.

### Sample size calculation

According to Tsuyuki et al., the incidence of nab-paclitaxel-induced sensory PN in hands treated with surgical glove compression therapy was approximately 28.1% (21.4/76.1) of the incidence in control hands [8]. Therefore, we set the incidence of patients wearing two normal-sized gloves on both hands to approximately 30% of that of the control group at a significance level of *p* < 0.025 (one-sided), with a statistical power of 0.9 and an estimated sample loss of 10%. The planned sample size is 104 patients (52 per group).

### Statistical analysis

The rate of change of the quantitative variable will be calculated by subtracting the initial value from the final value and dividing the result by the initial value. Missing values will not be replaced with other values. All efficacy analyses will be conducted using the intention-to-treat and per-protocol sets. The percentage of patients with a reduction of at least 10% or 5 points in the FACT-Taxane NTX score after using either two normal-sized gloves on both hands (intervention) or nothing (control) will be compared using a chi-square test. In addition, for each variable (including the FACT-Taxane score), the percentage change after 12 weeks of using two normal-sized gloves on both hands or nothing will be analyzed. The demographic characteristics of patients wearing either two normal-sized gloves on both hands (intervention) or nothing (control) will be summarized using the mean (standard deviation) for continuous data by group and count (percentage) for categorical data. Comparisons between the treatment groups will be performed using the chi-square test (categorical variables) and two-sample t-tests (continuous variables). All statistical analyses will be performed using the STATA software (version 16.0; StataCorp LP, College Station, TX, USA). *p* < 0.05 will be considered to be statistically significant.

### Randomization and data collection

Participants will be randomly assigned to the intervention and control arms in a 1:1 allocation ratio. Stratified randomization will be performed by the participating centers. The assignment list will be generated using a computer program, and investigators will be notified through an electronic case report from the clinical trial site at the time of enrollment (https://www.ecrf.kr/aiur). Investigators at each participating hospital will collect the required medical information to ensure accuracy. Data will be stored in an online electronic case report form with private information replaced by identification numbers. Each investigator will have access to the website and private account, and all data will be accessible only to the principal investigator.

### Safety monitoring

In this study, we will assess and gather adverse event data according to NCI-CTCAE version 5.0 and then perform a causality assessment. Severe adverse events will be reported to the principal investigator within 24 h after primary recognition of the event by the researcher. Likewise, severe adverse events will also be reported to the IRB within the time frame designated by the IRB guidelines. Medical issues directly related to the events that may occur during this clinical trial will be compensated for in accordance with the clinical research agreement and insurance.

## Discussion

This randomized controlled trial is designed to evaluate the efficacy of compression therapy using normal-sized surgical gloves to prevent CIPN in patients with breast cancer receiving paclitaxel treatment. We believe the results of this study could potentially provide valuable evidence for a practical, cost-effective, and easily accessible intervention for CIPN prevention. The primary outcome will be assessed using the FACT-Taxane NTX component questionnaire—a patient-reported outcome measure that has gained increasing importance in clinical trials to accurately capture patients’ experiences and symptoms. Additionally, the multicenter study design may increase the generalizability of the results.

CIPN typically presents as a symmetric, distal, stocking-and-glove distribution. However, the current intervention targets the upper extremities, thereby limiting preventive effects on the lower extremities. Thus, the assessment of generalized QoL and systemic symptoms may compromise our expected results. Nevertheless, studies have shown that after completing the paclitaxel treatment, approximately 50% of patients exhibit improvements within a 4–6-month period [31, 32]. Consequently, this study will include follow-up assessments up to 6 months after chemotherapy. Furthermore, CIPN primarily involves sensory rather than motor symptoms, even though Hongnan et al. suggested that the risk of motor and autonomic symptoms is common with taxanes. To explain this discrepancy, we hypothesized that this might be due to the use of steroids as premedication since steroid-associated myopathy is the most frequent cause of muscle disorders in patients with cancer [33].

In many cases, CIPN naturally improves over time following the cessation of chemotherapy; however, severe neuropathy can persist. For instance, Hershman et al. reported that up to 80% of patients in their study experienced neuropathic symptoms for up to 2 years after treatment [31].

Surprisingly, unlike chemotherapy-induced nausea and vomiting, the prevention of CIPN is not widespread. As PN can reduce treatment adherence and cause long-term issues, prevention should be considered during chemotherapy that can induce PN, regardless of the risk. However, no clear recommendation for CIPN prevention is currently available. Cryotherapy using frozen gloves showed promising results in small studies, and some neuropathy symptoms were alleviated [34, 35]. However, in the largest randomized phase III study, no differences in the CIPN subscales using the European Organization for Research and Treatment of Cancer CIPN 20-item scale were reported, with a notably high discontinuation rate due to discomfort [11]. Therefore, we suggest that if the results of this current study are significant and reveal few side effects or discomfort, they should be cautiously recommended for routine prevention of CIPN.

## Conclusion

This intervention is easily applicable in clinical practice and may serve as a preventive measure for CIPNs with strong patient adherence. Furthermore, if successful, this intervention is expected to improve QoL and treatment adherence in patients receiving chemotherapy that can induce PN beyond paclitaxel treatment alone.

## Supplementary Information

**Additional file 1. **

## Data Availability

Not applicable as no datasets were generated or analyzed during the current study. All the procedures were followed in accordance with the Declaration of Helsinki in the Ethics approval and consent to participate.

## Abbreviations

CIPN: Chemotherapy-induced peripheral neuropathy
QoL: Quality of life
PN: Peripheral neuropathy
FACT-NTX: Functional Assessment of Cancer Therapy Neurotoxicity
NCI-CTCAE: National Cancer Institute’s Common Terminology Criteria for Adverse Events
IRB: Institutional Review Board
IV: Intravenous
FACT-G: FACT-General
