# Supplementary material for: A randomized controlled trial using surgical gloves to prevent chemotherapy-induced peripheral neuropathy by paclitaxel in breast cancer patients (AIUR trial)
Source: BMC Cancer. 2023 Jun 20;23:570. doi: 10.1186/s12885-023-11079-8 (PMC10283240; doi:10.1186/s12885-023-11079-8)
Supplement: Supplementary file 1 — Additional file 1. [file 12885_2023_11079_MOESM1_ESM.docx]

|  | **PHYSICAL WELL-BEING** | **Not at all** | | **A little bit** | **Some-what** | **Quitea bit** | **Very much** |
| --- | --- | --- | --- | --- | --- | --- | --- |
|  |  |  |  |  |  |  |  |
| GP1 | I have a lack of energy | 0 | | 1 | 2 | 3 | 4 |
| GP2 | I have nausea | 0 | | 1 | 2 | 3 | 4 |
| GP3 | Because of my physical condition, I have trouble meeting the needs of my family | 0 | | 1 | 2 | 3 | 4 |
| GP4 | I have pain | 0 | | 1 | 2 | 3 | 4 |
| GP5 | I am bothered by side effects of treatment | 0 | | 1 | 2 | 3 | 4 |
| GP6 | I feel ill | 0 | | 1 | 2 | 3 | 4 |
| GP7 | I am forced to spend time in bed | 0 | | 1 | 2 | 3 | 4 |
|  | | | | | | | |
|  | **SOCIAL/FAMILY WELL-BEING** | **Not at all** | | **A little bit** | **Some-what** | **Quitea bit** | **Very much** |
|  |  |  |  |  |  |  |  |
| GS1 | I feel close to my friends | 0 | | 1 | 2 | 3 | 4 |
| GS2 | I get emotional support from my family | 0 | | 1 | 2 | 3 | 4 |
| GS3 | I get support from my friends | 0 | | 1 | 2 | 3 | 4 |
| GS4 | My family has accepted my illness | 0 | | 1 | 2 | 3 | 4 |
| GS5 | I am satisfied with family communication about my illness | 0 | | 1 | 2 | 3 | 4 |
| GS6 | I feel close to my partner (or the person who is my main support) | 0 | | 1 | 2 | 3 | 4 |
| Q1 | *Regardless of your current level of sexual activity, please answer the following question. If you prefer not to answer it, please mark this box and go to the next section.* | |  |  |  |  |  |
| GS7 | I am satisfied with my sex life | | 0 | 1 | 2 | 3 | 4 |

|  | **EMOTIONAL WELL-BEING** | **Not at all** | | **A little bit** | **Some-what** | **Quitea bit** | **Very much** |
| --- | --- | --- | --- | --- | --- | --- | --- |
|  |  |  |  |  |  |  |  |
| GE1 | I feel sad | | 0 | 1 | 2 | 3 | 4 |
| GE2 | I am satisfied with how I am coping with my illness | | 0 | 1 | 2 | 3 | 4 |
| GE3 | I am losing hope in the fight against my illness | | 0 | 1 | 2 | 3 | 4 |
| GE4 | I feel nervous | | 0 | 1 | 2 | 3 | 4 |
| GE5 | I worry about dying | | 0 | 1 | 2 | 3 | 4 |
| GE6 | I worry that my condition will get worse | | 0 | 1 | 2 | 3 | 4 |

|  | **FUNCTIONAL WELL-BEING** | **Not at all** | | **A little bit** | **Some-what** | **Quitea bit** | **Very much** |
| --- | --- | --- | --- | --- | --- | --- | --- |
|  |  |  |  |  |  |  |  |
| GF1 | I am able to work (include work at home) | | 0 | 1 | 2 | 3 | 4 |
| GF2 | My work (include work at home) is fulfilling | | 0 | 1 | 2 | 3 | 4 |
| GF3 | I am able to enjoy life | | 0 | 1 | 2 | 3 | 4 |
| GF4 | I have accepted my illness | | 0 | 1 | 2 | 3 | 4 |
| GF5 | I am sleeping well | | 0 | 1 | 2 | 3 | 4 |
| GF6 | I am enjoying the things I usually do for fun | | 0 | 1 | 2 | 3 | 4 |
| GF7 | I am content with the quality of my life right now | | 0 | 1 | 2 | 3 | 4 |

|  | **ADDITIONAL CONCERNS** | **Not at all** | | **A little bit** | **Some-what** | **Quite**  **a bit** | **Very much** |
| --- | --- | --- | --- | --- | --- | --- | --- |
|  |  |  |  |  |  |  |  |
| NTX1 | I have numbness or tingling in my hands | | 0 | 1 | 2 | 3 | 4 |
| NTX2 | I have numbness or tingling in my feet | | 0 | 1 | 2 | 3 | 4 |
| NTX3 | I feel discomfort in my hands | | 0 | 1 | 2 | 3 | 4 |
| NTX4 | I feel discomfort in my feet | | 0 | 1 | 2 | 3 | 4 |
| NTX5 | I have joint pain or muscle cramps | | 0 | 1 | 2 | 3 | 4 |
| HI12 | I feel weak all over | | 0 | 1 | 2 | 3 | 4 |
| NTX6 | I have trouble hearing | | 0 | 1 | 2 | 3 | 4 |
| NTX7 | I get a ringing or buzzing in my ears | | 0 | 1 | 2 | 3 | 4 |
| NTX8 | I have trouble buttoning buttons | | 0 | 1 | 2 | 3 | 4 |
| NTX9 | I have trouble feeling the shape of small objects when they are in my hand | | 0 | 1 | 2 | 3 | 4 |
| An6 | I have trouble walking | | 0 | 1 | 2 | 3 | 4 |
| Tax1 | I feel bloated | | 0 | 1 | 2 | 3 | 4 |
| Tax2 | My hands are swollen | | 0 | 1 | 2 | 3 | 4 |
| Tax3 | My legs or feet are swollen | | 0 | 1 | 2 | 3 | 4 |
| Tax4 | I have pain in my fingertips | | 0 | 1 | 2 | 3 | 4 |
| Tax5 | I am bothered by the way my hands or nails look | | 0 | 1 | 2 | 3 | 4 |
